# A Review of Alternative Treatment Options in Diabetic Polyneuropathy

**DOI:** 10.7759/cureus.14600

**Published:** 2021-04-21

**Authors:** Arsalan Zaheer, Faizan Zaheer, Hadia Saeed, Zoya Tahir, Muhammad Waqas Tahir

**Affiliations:** 1 Medicine, Gujrat Hospital, Gujrat, PAK; 2 Medicine, Fatima Memorial Hospital College of Medicine and Dentistry, Lahore, PAK; 3 Pathology, Shaikh Zayed Hospital, Lahore, PAK; 4 Internal Medicine, Rochester General Hospital, Rochester, USA

**Keywords:** diabetes, diabetic polyneuropathy, non-pharmacological treatments, natural supplements

## Abstract

Currently there is no recognized curative treatment for diabetic polyneuropathy (DPN). Strict glucose control and symptomatic pain relief are the first line management routes. DPN is a common complication of diabetes and has a major detrimental influence on the quality of life (QOL) for many patients. Due to the scope of the problem, it is imperative that treatment options which impede DPN's progression and restore sensorineural function should be researched comprehensively and made available to the masses at an economical cost. We reviewed a multitude of atypical treatment options for DPN including capsaicin, lidocaine, acupuncture, electrical nerve stimulation, alpha lipoic acid, benfotiamine, and acetyl-l-carnitine and explored the evidence to date regarding their safety and efficacy. Most of these options have been around for a long time and have promising pilot studies or small-scale trials focused on DPN treatment.

## Introduction and background

Diabetes is the ninth major cause of death globally, and in the past three decades, the number of diabetic patients has quadrupled. Diabetes affects people of all ethnicities, ages, and genders [1]. Diabetes mellitus has two identified subtypes: type 1 and type 2. Type 1 begins by progressive autoimmune-mediated loss of pancreatic β-cell mass due to an environmental trigger causing a complete absence of insulin [2]. Type 2, on the other hand, is caused by decreased insulin sensitivity due to multiple genetic and environmental factors, which eventually lead to a reduction in B-cell functioning [3]. Diabetic peripheral neuropathy is the most common complication of diabetes, affecting 50% of patients [4]. Multiple metabolic processes have been implicated in the development of diabetic polyneuropathy (DPN). Hyperglycemia, elevated homocysteine levels, reduced nitric oxide, excessive production of reactive oxygen species (ROS), and increased sorbitol and protein kinase C damage endothelial tissue, creating an irreversible change by increasing vascular resistance and decreasing blood supply to the nerves resulting in neuropathy [5].

The diagnosis of DPN is through exclusion through a combination of focused investigations and the detailed clinical assessment of the neuropathic symptoms. Neuropathic symptoms could be commonly present with normal investigative results. Symptoms for DPN vary depending on the nerves affected and develop gradually over many years [5]. Pain in the extremities is the most troublesome sign of DPN, usually described as a burning or stabbing sensation, and it can be accompanied by numbness, hyperesthesia or a deep ache. DPN and its complications cause considerable mortality, morbidity and deterioration in the quality of life (QOL) [6].

The prevention and management strategies of DPN traditionally focus on glycemic control, and hypoglycemics and dietary control play a significant role. Ideal management of factors such as hypertension, hypercholesterolemia, and hyperglycemia delay DPN development [5]. Nevertheless, even with sufficient control of these risk factors, over 40% patients still develop DPN [7-8]. There are multiple symptomatic relief strategies for DPN; however, there is no identifiable treatment course that halts or impedes disease progression. The treatment options can broadly be categorized as pharmacological and non-pharmacological/topical modalities. The former includes antidepressants and gabapentinoid antiepileptics. These agents are well studied and have proven benefit in symptom control. The non-pharmacological or topical treatment methods are diverse including nutritional supplements like benfotiamine, alpha-lipoic acid, etc.; topical treatments like capsaicin, lidocaine patch; and neuromodulation treatments like electrical nerve stimulation. The treatments in this diverse group are either poorly studied or even if studied well with proven effects, are underutilized. There have been several trials on these modalities which have shown promise in decreasing the severity of DPN. Herein, we review the evidence of efficacy of these atypical treatment options for DPN.

## Review

Capsaicin

Found primarily in chili peppers, capsaicin works by targeting the nociceptor to produce pain. However, high or repetitive doses of capsaicin induces an initial pain sensation that is followed by analgesia [9].

Over the years, the idea of repetitive doses of capsaicin has been translated into higher concentration application. Early trials focused on low concentration capsaicin. A clinical trial from 1992 assessed the therapeutic benefits of topical 0.075% capsaicin in patients with chronic severe painful DPN, who were unresponsive to conventional treatment. Twenty-two patients were randomly assigned to be treated with capsaicin or a placebo cream over painful areas four times a day, over the course of eight weeks. Pain measures were assessed by baseline, two weeks and at eight weeks. The trial established that 0.075% capsaicin was more effective than placebo in decreasing pain intensity. During the follow-up open-label study, 50% of the patients reported improved pain control, whereas 25% reported that pain being unchanged or worse [10]. More recent studies on capsaicin are focused on higher concentration. A 12-week clinical trial evaluated the efficacy and safety of capsaicin 8% patch versus placebo patch in painful DPN. A total of 369 patients were randomized to a one 30-minute treatment (capsaicin 8% patch or placebo patch) to painful areas of the feet. The trial showed that patients with painful DPN treated with 8% capsaicin experienced moderated pain relief from week-two onwards and overall improvement in sleep quality when compared to the placebo group. There were no systemic side effect or neurosensory deterioration observed with high concentration capsaicin [11].

The evidence seems to be in favor of capsaicin’s therapeutic effect and safety but there is lack of active agent control trials for capsaicin and no consensus on the optimal concentration. Nevertheless, American Academy of Neurology, the American Association of Neuromuscular and Electrodiagnostic Medicine, and the American Academy of Physical Medicine and Rehabilitation consider capsaicin as a probably effective in lessening DPN symptoms and give it a level B recommendation [12]. Further studies comparing capsaicin to systemic treatments, other topical modalities and different strengths of capsaicin would provide more insight into its efficacy.

Lidocaine patches

Lidocaine was first manufactured in 1943. It functions through binding with sodium channels responsible for the generation of action potential, thereby preventing the transmission of impulses in the pain nerve fibers causing local anesthesia [13].

An open-label, three-week trial was conducted to gauge the efficiency and tolerability of the 5% lidocaine patch in painful DPN. The dosing was flexible allowing up to four lidocaine patches for up to 18 hours daily. Patients showed significant improvements in pain and QOL measures during the three-week treatment period. A subgroup of patients treated for an additional five weeks retained the therapeutic benefits which allowed for the tapering of concomitant analgesic therapy. Systemic accumulation of lidocaine was not observed and the adverse effects were minimal [14].

A two-week, open-label, nonrandomized, multicenter pilot trial was conducted to assess the efficacy and safety of the lidocaine patch 5% in patient with postherpetic neuralgia, DPN, and low back pain. Forty-nine of 107 included patients had DPN. Efficacy was assessed using the brief pain inventory (BPI). The trial concluded that with two weeks use of lidocaine patch treatment, all disease groups reported significant improvements of pain intensity and decrease in BPI scores. There were no reported side effects [15].

Randomized controlled trials with placebo as well as other active agents are needed to rank its efficacy compared to first line agents.

Electrical nerve stimulation

Transcutaneous electrical nerve stimulation (TENS) was developed in 70s as a measure for pain tolerance in patient with chronic pain. Today it is used as a non-invasive method of pain relief through regulated electrical stimuli. It functions by sending an electrical pulse across intact skin to stimulate underlying nerves. TENS generates a non-painful electrical stimulus to elicit paresthesia [16].

A randomized controlled trial involving 31 DPN patients was conducted to assess efficacy of TENS as a non-pharmacological treatment modality. Patients treated their lower extremities for 30 minutes each day for four weeks at home. Five patients from the placebo group and 15 patients from the active therapy group reported clinically significant reduction in pain scores. However, the active electrotherapy group reported substantial therapeutic effect far above the pain scores from the placebo group. The trial concluded that TENS reduced pain and discomfort of painful DPN [17]. One placebo controlled single blinded randomized study consisting of 41 DPN patients was undertaken to assess the therapeutic extent of micro-TENS therapy in reducing neuropathic pain. Twenty-two patients were treated with micro-TENS and 19 were treated with placebo therapy. Trial lasted for four weeks with three therapy session per week. Standardized questionnaires were used to assess pain intensity, pain disability, and QOL at baseline at the end of the treatment period. Six patients in the active therapy group and 10 patients in the placebo group reported improvement in pain intensity. The trail concluded that micro-TENS was not superior to placebo in the treatment of neuropathy [18].

In a double blind, randomized study, 19 DPN patients received either treatment with a low frequency stimulation TENS device and placebo treatment with a duplicate but an inactive device. The patient’s symptoms were recorded at baseline, at six weeks and at 12 weeks of treatment using the new total symptom score (NTSS-6) and a visual analogue scale (VAS). Sensory nerve thresholds (temperature, vibration, pain) and microvascular function were measured at baseline and after 12 weeks of treatment. The trial concluded that TENS significantly improved pain scores, numbness and allodynia. There were no changes recorded in the placebo arm [19].

Trial results using TENS for pain control in DPN have yielded mixed results. TENS has also been studied for several other neuropathic pain syndromes like post-herpetic neuralgia, chronic low back pain, etc., and the results show equivocal to no benefit. For DPN, studies are small in size with no standard type of TENS studied. Bigger studies comparing TENS to other topical treatment options, systemic therapies and comparing different TENS device types are needed to prove its therapeutic effects. American Academy of Neurology considers it probably effective for treatment of DPN [20].

Acupuncture

One of the oldest non-pharmacological treatments for pain is acupuncture, which dates back to 100 C.E. The first-time acupuncture was studied in US was in the 70s. Acupuncture has various possible mechanism of action, however, the most supported theory is the release endogenous opioids, causing an analgesic effect [21].

There are handful of studies focusing on use of acupuncture for pain control in DPN. A small trial involving 46 DPN patient was carried to gauge the safety and efficacy of acupuncture. Patients were treated six times over 10 weeks. Thirty-four patients showed improvement in their symptom. The patients were followed for the next 18-52 weeks, 67% of the patients were able to either stop or significantly reduce their analgesic medication. A total of 77% reported clinically significant improvement in their symptoms, whereas 21% reported complete cessation of their symptoms. The trial concluded that acupuncture is a safe and effective treatment for the long-term management of painful DPN [22]. Another single-blinded, placebo-controlled trial was conducted to assess the treatment benefit of acupuncture for DPN patients. Forty-five patients were enrolled to receive either placebo or active treatment. The trial lasted for 10 weeks. This trial too concluded that acupuncture was safe and should be considered a possible option for adjuvant therapy for DPN patients [23].

A pilot study evaluated the therapeutic significance of two different methods of acupuncture: Traditional Chinese Medicine (TCM) and Japanese acupuncture in the treatment of painful DPN. The study lasted 10 weeks and consisted of seven patients only. Treatments were delivered once a week for trial duration. The trial concluded that patients assigned to Japanese acupuncture reported decreased pain intensity, whereas the group treated with TCM acupuncture reported minimal effects when measured using daily pain severity score. However, both methods reduced pain intensity per McGill Short Form Pain Score [24]. A randomized clinical trial evaluated the feasibility, acceptability, and effects of group acupuncture for DPN. Trial enrolled 40 patients randomized to two active groups and a placebo group for treatment over 12 weeks. The trial determined that acupuncture did produce clinically significant pain reduction and improvement in QOL [25].

Similar shortcomings are seen in evidence for efficacy of acupuncture in DPN as with other topical modalities. Studies are either very small in size or are just pilot studies without a follow-up larger study even if there is a signal of effect in smaller study. Moreover, the methodology in most trials is questionable. A meta-analysis consisting of 25 trials involving 1,649 participants although showed that acupuncture when compared to treatment with B12, B1, mecobalamin and no treatment had a better effect on general symptoms, the authors pointed out that clinically relevant conclusions cannot be drawn owing to poor design of most included trials and trials’ high risk of bias. For acupuncture to be considered as treatment option for DPN, better designed studies with relevant comparison arms are needed to show its benefits if any.

Alpha-lipoic acid

Alpha-lipoic acid (ALA) is a biological nutrient produced in minute quantities by plants and animals, including humans. ALA functions as an antioxidant and has a chemoprotective nature, as it oxidizes free radical and inhibits the degeneration of tissue cells. ALA concurrently plays the role of a cofactor for multiple mitochondrial enzyme interactions and can effectively regenerate other antioxidants such as vitamin C, vitamin E, and glutathione through redox cycling [26-28]. Multiple clinical trials and meta-analysis have been conducted to evaluate ALA as a treatment option.

A meta-analysis of four randomized, double blinded, placebo-controlled trials comprising of 1258 patients assessed the safety and the therapeutic value of intravenous (IV) ALA. The study concluded lipoic acid (600 mg daily) over three weeks as safe and quantitatively exhibited significant improvement in neuropathic symptoms [29]. A multicenter, randomized, double-blind, placebo-controlled trial with populations of 181 diabetic patients with DPN were treated with oral ALA (600 mg to 1800 mg daily) for six weeks. This trial showed improved neuropathic symptoms and neural deficits in patients with DPN at the end of treatment period [30]. A multicenter open-label trial of 45 patients studied ALA in step-wise dose. All participants received initial four-week high-dose (600 mg TID) of ALA, with subsequent treatment of phase I responders with low-dose ALA (600 mg daily) for next 16 weeks. The responders had significantly diminished neuropathic symptoms at the end of study [31]. The Nathan 1 trial was a long term, a multicenter randomized, double-blind parallel-group trial, which included 460 diabetic patients with mild-to-moderate diabetic symmetrical polyneuropathy (DSPN). It was conducted over four years to gauge the long-term safety and efficacy of ALA. Use of ALA was safe and associated with improvement of neuropathic impairment scores. Nerve conduction scores were similar in ALA and placebo groups [32]. A retrospective study of 443 patients on long-term ALA (mean length of treatment = 5 years), who were switched to central analgesic (mainly gabapentin), showed considerably higher rates of side effects, frequencies of outpatient visits, and daily costs of treatment in central analgesic group as compared to ALA.

Based on the current evidence, ALA use is associated with clinically significant improvement in neuropathic symptoms, with pain being the most improved symptom and can be considered as a treatment modality. Head-to-head studies comparing ALA to first-line pharmacotherapeutic agents such as gabapentin are needed.

Acetyl-L-Carnitine

Acetyl-L-Carnitine (ALCAR/ALC) is the derived amino acid form, produced by the acetylating carnitine [33]. It serves as a cofactor facilitating acetyl-CoA's movement into the mediums of human mitochondria in energy generation from fatty acid oxidation. In addition to its metabolic role, acetyl-L-carnitine has neuroprotective, neuromodulatory, and neurotrophic properties. It has been shown to promote peripheral nerve health by increasing nerve conduction velocity (NCV), reducing sensorineural loss, and supporting nerve regeneration [34]. Simultaneously ALC stimulates an analgesic effect in patients with HIV-related, chemotherapy-induced, and diabetic neuropathies [35].

A randomized, double-blind, placebo-controlled clinical trial involving 333 patients was conducted over one year to assess ALC's therapeutic value. ALC was administered intramuscularly (1 g daily) for 10 days followed by oral dosage of 2 g daily for the remaining period of 355 days. Nerve conductions studies at conclusion compared to baseline showed significant electrophysiological as well as pain score improvement with ALC compared to the placebo group [36]. An analysis of two randomized placebo-controlled trials involving 1257 patients assessed the safety and the therapeutic value of ALC. The two clinical trials tested the effects of two doses of 500 mg and 1000 mg TID. Electrophysiological parameters at baseline incorporated sural nerve morphometry, NCV, and vibrations perception thresholds. Concomitantly clinical symptoms scores and VAS for pain intensity assessment measures were used to compare neurophysiological changes from the baseline. In regards to development, both trials showed improvements in electrophysiological parameters such as sural nerve fiber quantities and the regeneration of nerve fiber clusters as well as clinical parameters such as VAS scores. Higher dose was associated with better outcome in clinical pain parameters [37].

A randomized, double-blind, multicenter clinical trial was conducted to demonstrate the safety and efficacy of ALC to methylcobalamin (MC) in the treatment of diabetic neuropathy. A total number of 232 diabetic patients were randomized to receive ALC or MC for 24 weeks. During the trial, clinical progress was assessed by the neuropathic symptom score, the neuropathy disability score, and the neurophysiological parameters. Each group at week 24 showed a noteworthy decline in both neuropathy symptom score and neuropathy disability score with no noted significant difference between the two groups [38].

These clinical trials demonstrate safety and efficacy of ALC in treating DPN. The improvement in electrophysiological measures shows a more objective evidence of potential therapeutic benefit of ALC in DPN. There are no trials comparing ALC to standard-of-care treatment for DPN. Furthermore, the long-term data in placebo-controlled trials is restricted to one year only. Hence, the quality of evidence is still not considered high enough for it to be used for treatment of DPN as an alternative or adjunct to the pharmacologic agents.

Benfotiamine

Developed in late 1950s in Japan, benfotiamine is a synthetic lipid form of thiamin which is used as a treatment option for alcoholic neuropathy, sciatica, and other painful nerve disorders in alternative medicine. The mechanism for pain control is thought to be through increasing intracellular thiamine diphosphate levels, which serves as a cofactor of transketolase; this enzyme inhibits advanced glycation and lipid-oxidation end products, eventually preventing microvascular complications in diabetics [39].

A randomized pilot study was conducted to evaluate the efficacy of benfotiamine in patients with DPN. The study included 40 patients with type 1 and type 2 diabetes with two or more years of neuropathic symptoms. One group received 50 mg of benfotiamine four times daily, and another group received a placebo. The study lasted for three weeks. A neuropathy pain score was used to assess symptoms of polyneuropathy and vibration threshold was used as electrophysiological baseline parameter. The trial concluded a significant improvement in the neuropathic score in the benfotiamine treated group compared to the placebo group. However, no changes were reported in regards to improvements in vibration perception threshold [40]. A double-blind, placebo-controlled, phase-III-study including 165 patients with DPN were assigned to three groups, i.e., benfotiamine 600 mg daily, benfotiamine 300 mg daily and placebo. After six weeks of treatment, the neuropathic symptoms score showed improvement with benfotiamine compared to placebo with more improvement in higher dose group [41].

Evidence for benfotiamine in treatment of DPN is still poor primarily due to few high-quality studies and short follow-up period. Moreover, there are no head-to-head trials with first line treatments for DPN. It is an economical and readily available product and could potentially serve as an adjuvant supplement for restoring normal physiological nerve function in diabetics. It may help with other chronic complications of diabetes due to its presumed mechanism of action.

## Conclusions

Treatment modalities other than the typical pharmacologic agents used for painful diabetic peripheral neuropathy have been around for a long time. Their use in general practice particularly for DPN is limited. Safety data for the use of these topical treatments (capsaicin and lidocaine), neuromodulation therapies (acupuncture and TENS) or nutritional supplements (ALA, ALC and benfotiamine) is reasonably convincing. Efficacy data for some is more convincing than others. Almost all show significantly better symptom (primarily pain) control consistently in small to big studies. Those with better study designs (randomized placebo-controlled trials with good sample size and considerable follow-up periods) like capsaicin and ALA can be considered as an adjunct to DPN treatment. Most of these modalities lack evidence where they are compared to first-line therapeutic options. In absence of such evidence, despite their seemingly efficacious results from the pilot studies, they fail to make it into mainstream treatment guidelines.

Further studies are needed to explore these potential treatment options. One or more of these options could potentially be equally good if not better than the typical pharmacologic agents used to treat DPN and may have more value because of their cost benefit and benign side effect profiles. Studies to identify optimal dosing and formulation of these therapies especially contrasting them to the typical agents with long-term follow-up data would help create better treatment alternatives for this globally ubiquitous problem.
